# Collapsin Response Mediator Protein 2 (CRMP2) Modulates Mitochondrial Oxidative Metabolism in Knock-In AD Mouse Model

**DOI:** 10.3390/cells14090647

**Published:** 2025-04-29

**Authors:** Tatiana Brustovetsky, Rajesh Khanna, Nickolay Brustovetsky

**Affiliations:** 1 Department of Pharmacology and Toxicology, Indiana University School of Medicine, Indianapolis, IN 46202, USA; tbrousto@iu.edu; 2 Department of Pharmacology & Therapeutics, University of Florida College of Medicine, Gainesville, FL 32610, USA; r.khanna@ufl.edu; 3 Center for Advanced Pain Therapeutics and Research (CAPToR), University of Florida College of Medicine, Gainesville, FL 32610, USA; 4 Stark Neurosciences Research Institute, Indiana University School of Medicine, Indianapolis, IN 46202, USA

**Keywords:** CRMP2, Alzheimer’s disease, cortical neurons, mitochondrial oxidative metabolism, mitochondrial ROS production, adenine nucleotide translocase

## Abstract

We explored how the phosphorylation state of collapsin response mediator protein 2 (CRMP2) influences mitochondrial functions in cultured cortical neurons and cortical synaptic mitochondria isolated from APP-SAA KI mice, a knock-in APP mouse model of Alzheimer’s disease (AD). CRMP2 phosphorylation was increased at Thr 509/514 and Ser 522 in brain cortical lysates and cultured neurons from AD mice. The basal and maximal respiration of AD neurons were decreased. Mitochondria were hyperpolarized and superoxide anion production was increased in neurons from AD mice. In isolated synaptic AD mitochondria, ADP-stimulated and DNP-stimulated respiration were decreased, whereas ADP-induced mitochondrial depolarization was reduced and prolonged. We found that CRMP2 binds to the adenine nucleotide translocase (ANT) in a phosphorylation-dependent manner. The increased CRMP2 phosphorylation in AD mice correlated with CRMP2 dissociation from the ANT and decreased ANT activity in AD mitochondria. On the other hand, recombinant CRMP2 (rCRMP2), added to the ANT-reconstituted proteoliposomes, increased ANT activity. A small molecule (S)-lacosamide ((S)-LCM), which binds to CRMP2 and suppresses CRMP2 phosphorylation by Cdk5 and GSK-3β, prevented CRMP2 hyperphosphorylation, rescued CRMP2 binding to the ANT, improved ANT activity, and restored the mitochondrial membrane potential and respiratory responses to ADP and 2,4-dinitrophenol. Thus, our study highlights an important role for CRMP2 in regulating the mitochondrial oxidative metabolism in AD by modulating the ANT activity in a phosphorylation-dependent manner.

## 1. Introduction

Alzheimer’s disease (AD) is a devastating, incurable neuropathology associated with neuronal dysfunction and memory impairment. AD is a frequent cause of dementia and a major contributor to illness and death in elderly individuals. In Alzheimer’s disease, one of the initial signs of brain pathology is the deterioration of neurons. This degeneration is probably caused by the accumulation of β-amyloid plaques and neurofibrillary tangles. The breakdown of neural structures, including impaired neuron function and the loss of synaptic connections, can lead to problems with cognition and memory. The mechanisms leading to neuronal dysfunction and synaptic defects in AD are not entirely clear.

Mitochondrial defects are a significant factor in AD pathology [1,2,3,4,5,6,7,8,9,10,11,12,13,14,15,16]. The mitochondrial oxidative metabolism plays a crucial role in satisfying energy demands in neurons [17,18,19]. In Alzheimer’s disease, changes in mitochondrial energy production and structural abnormalities have been observed. These alterations are thought to play a role in synaptic issues and neuron breakdown [2,3,4,5,11,20,21,22]. Despite these findings, the precise processes causing mitochondrial energy deficits in Alzheimer’s remain unclear.

Mitochondrial bioenergetics relies on the oxidative phosphorylation (OXPHOS) system. Mitochondrial F_1_F_0_-ATP synthase (ATP synthase) is a major component of the OXPHOS system [23], while adenine nucleotide translocase (ANT) is a key transporter in the inner mitochondrial membrane (IMM), exchanging cytosolic ADP for mitochondrial ATP [24,25]. The activities of ATP synthase and the ANT are reduced in AD [26,27,28,29,30], but the mechanisms contributing to this reduction are not entirely clear.

Collapsin response mediator protein 2 (CRMP2) is an abundant cytosolic phosphoprotein originally implicated in the regulation of neurite outgrowth [31], and later in AD pathology [32,33,34,35,36]. CRMP2 serves as a physiological target for the kinases glycogen synthase kinase-3β (GSK-3β) and cyclin-dependent kinase 5 (Cdk5), both of which show heightened activity in Alzheimer’s disease [37,38,39,40]. CRMP2 phosphorylation at the residues targeted by GSK-3β and Cdk5 is higher in human AD brains [41,42,43,44,45] and in the brains of mouse models of AD [43,45,46]. In AD mice, CRMP2 hyperphosphorylation was reported as early as 2 month of age and, thus, occurs prior to pathology, suggesting that increased CRMP2 phosphorylation is an early event in AD progression [43]. However, the functional consequences of CRMP2 hyperphosphorylation and their importance for AD pathology have not been investigated in detail.

CRMP2 binds to mitochondria [47,48]. A fraction of CRMP2 resides in the intermembrane space between the inner and the outer mitochondrial membranes [48], and interacts with the adenine nucleotide translocase 1 [47], a key player in mitochondrial bioenergetics [24]. CRMP2 hyperphosphorylation is paralleled by the dissociation of CRMP2 from the ANT [47]. CRMP2 hyperphosphorylation in AD [41,42,43,44,45,46] implies that, in AD, CRMP2 may dissociate from the ANT. The consequences of CRMP2 dissociation from the ANT for mitochondrial oxidative metabolism in AD are not clear. It is also unclear whether preventing CRMP2 hyperphosphorylation and preserving CRMP2 interaction with the ANT are beneficial for mitochondrial oxidative metabolism.

Here, we investigated the effect of CRMP2 phosphorylation state on the mitochondrial oxidative metabolism in APP-SAA KI mice, a knock-in AD mouse model [49]. We found increased CRMP2 phosphorylation in brain tissue lysates and in cultured cortical neurons from APP-SAA KI mice, which correlated with diminished CRMP2 binding to the ANT, decreased ANT activity, and altered mitochondrial respiration. The small molecule (S)-lacosamide ((S)-LCM) reduced CRMP2 hyperphosphorylation, restored CRMP2 binding to the ANT, rescued ANT activity, and improved respiration. Thus, in APP-SAA KI mice, CRMP2 affects mitochondrial oxidative metabolism in a phosphorylation-dependent manner by modulating ANT activity.

## 2. Materials and Methods

### 2.1. Animals

All animal procedures were conducted following the guidelines set by the US National Institutes of Health for laboratory animal care and use, and were approved by the Institutional Animal Care and Use Committee at Indiana University School of Medicine (#23156 MD/R/E). In our study, we used knock-in APP-SAA KI [49] (Jackson Laboratories, Bar Harbor, ME, USA, Strain # 034711), which carry humanized Aβ region R684H, F681Y, and G676R mutations, and the KM670/671NL (Swedish) mutation in exon 16 as well as the E693G (Arctic) and T714I (Austrian) mutations in exon 17 of the mouse *App* gene. As a control, we used B6J hAbeta mice (B6.Cg-App^em1Adiuj^/J, Strain #033013, Jackson Laboratory), which express APP with a humanized Aβ 1–42 region as in APP-SAA KI mice, but without any mutations. With both strains, mice of both sexes were used. Breeding colonies were housed at the Laboratory Animal Resource Center, Indiana University School of Medicine, Indianapolis, IN, USA. The APP-SAA KI mice are less prone to artifacts associated with overexpression of APP in transgenic mouse models of AD [49], and, therefore, in our experiments we focused on APP-SAA KI mice.

### 2.2. Mouse Oral Gavage

We used mouse oral gavage to deliver a vehicle (10 μL DMSO in 200 µm saline) or (S)-LCM dissolved in DMSO (10 mg/kg body weight in 0.2 mL saline). A half-milliliter (mL) syringe with a specialized 20-gauge needle for mouse oral gavage (FN-7910, Roboz Surgical Instrument Co., Gaithersburg, MD, USA) was used.

### 2.3. Neuronal Cell Culture

Mouse cortical neurons in culture were prepared from postnatal day 1 (P1) APP-SAA KI and B6J hAbeta mice according to IACUC approved protocol and methodologies published earlier [50]. For immunoblotting and co-immunoprecipitation (co-IP) experiments, neurons were grown at 200,000 cells per Petri dish (Ø 35 mm). For evaluation of mitochondrial membrane potential and superoxide anion production, neurons were grown at lesser density (10,000 cells) per glass bottom (Ø 10 mm) Petri dish to decrease likelihood of neuronal clumping. For all experiments with cells, 35 μg/mL uridine plus 15 μg/mL 5-fluoro-2′-deoxyuridine were injected to the dishes 24 h after plating to inhibit propagation of microglia. Cells were kept in the air with 5% CO_2_ at 37 °C in MEM with added 10% NuSerum (BD Bioscience, Bedford, MA, USA) and 27 mM glucose. Neuronal cultures were utilized in experiments at 12–14 day in vitro (12–14 DIV).

### 2.4. Isolation and Purification of Brain Synaptic Mitochondria

Cortical synaptic mitochondria were isolated and purified with discontinued 24/40% Percoll gradient as we described earlier [48]. The purity of the standard mitochondrial preparation produced with discontinued 24/40% Percoll gradient purification has been shown in our recent paper [48].

### 2.5. Immunoblotting

Cultured cortical neurons (12–14 DIV) were prepared for gel-electrophoresis as follows. Cells were homogenized in a buffer containing 50 mM Tris-HCl (pH 7.4), 150 mM NaCl, 1% NP-40, 0.1% SDS, 1 mM EDTA, and a cocktail of phosphatase and protease inhibitors (Roche, Indianapolis, IN, USA), Cat # 04906845001 and Cat # 04693124001). The homogenates were kept on ice for 30 min, then centrifuged at 100,000 g for 30 min. The pellet was discarded, and the supernatant was used for gel electrophoresis. Proteins were separated using Bis-Tris gels (4–12%, Invitrogen (Carlsbad, CA, USA), Cat # NP0335) with 20 µg of protein per lane. Subsequently, proteins were transferred to a Hybond-ECL nitrocellulose membrane (Amersham Biosciences (Piscataway, NJ, USA), Cat # RPN78D). Blots were blocked at 22 °C for 60 min. For phosphoprotein blotting, a solution of 5% BSA, Tris-HCl buffered saline (pH 7.2), and 0.15% Triton X-100 was used. For total protein blotting, 5% milk, phosphate-buffered saline (pH 7.2), and 0.15% Triton X-100 were used. After blocking, blots were incubated with various antibodies: rabbit anti-CRMP2 pTyr 32 (gift from Dr. Yoshio Goshima (Yokohama City University, Yokohama, Japan), 1:1500), sheep anti-CRMP2 pThr 509/514 (Kinasource (Dundee, Scotland, UK), Cat # PB-043, 1:1500), rabbit anti-CRMP2 pSer 522 (ECM Biosciences, Cat # CP2191, 1:1500), rabbit anti-CRMP2 pThr 555 (ECM Biosciences (Versailles, KY, USA), Cat # CP2251, 1:1500), rabbit anti-CRMP2 (Sigma, (Saint Louis, MO, USA), Cat # C2993, 1:1000), rabbit anti-ANT 1/2 (Proteintech, (Rosemont, IL, USA), Cat # 15997-1, 1:1000), rabbit anti-ATP5G1 (Boster Biological Technology, (Pleasanton, CA, USA), Cat # M32382, 1:1000), and mouse anti-GAPDH (Abcam, (Cambridge, MA, USA), Cat # ab9484, 1:2000). Blots were incubated with horseradish peroxidase-conjugated goat anti-mouse or goat anti-rabbit IgG (1:25,000 or 1:20,000, respectively) from Jackson ImmunoResearch Laboratories. They were then developed using Supersignal West Pico chemiluminescent reagents (Pierce, (Rockford, IL, USA), Cat # 32106). To determine the molecular masses of the bands, the Page Ruler Plus Prestained Protein Ladder (5 μL, Thermo Fisher, (Waltham, MA, USA), Cat # 26619) was used. The immunoblot images were inverted, and the integrated density of the bands was quantified after background subtraction with Adobe Photoshop 22.2.0.

### 2.6. Co-Immunoprecipitation

Using a Percoll gradient-fractionation method, we isolated brain cortical synaptic mitochondria from 4-month-old APP-SAA KI mice, both untreated and treated with 10 mg/kg body weight (S)-LCM via oral gavage for 7 days, or with a vehicle (10 µL DMSO in 0.2 mL saline via gavage for 7 days). As a control, we used mitochondria from age-matched B6J hAbeta mice. After isolation, mitochondria were lysed in a buffer containing 125 mM KCl, 3 mM KH_2_PO_4_, 0.5 mM MgCl2, 10 mM Hepes (pH 7.4), Proteinase Inhibitor Cocktail (Roche), 1% NP40, and 0.1% SDS. Lysates were purified by treating with Protein A/G agarose beads (Santa Cruz Biotechnology, (Dallas, TX, USA), Cat # sc-2002) for 2 h at 4 °C. The lysates were then incubated overnight with primary rabbit anti-CRMP2 antibody (Sigma, Cat # C2993, 1:1000) or rabbit anti-ANT 1/2 antibody (Proteintech, Cat # 15997-1, 1:1000) under gentle shaking at 4 °C, followed by incubation with Protein A/G agarose beads (Santa Cruz Biotechnology, Cat # sc-2002) for 2 h at 4 °C. For co-IP experiments with F1F0-ATP-synthase subunit c and CRMP2, we used anti-ATP5G1 rabbit monoclonal antibody (Boster Biological Technology, Cat # M32382, 1:1000). The immune-captured complexes were washed three times with lysis buffer before being heated at 70 °C in equal volumes of SDS loading dye (Invitrogen). Proteins were separated by electrophoresis using Tris-Acetate gels (3–8%, Invitrogen, Cat # EA0375BOX) with 20 µg of protein per lane. Samples were subjected to immunoblotting as previously described [51,52]. Blots were incubated with rabbit anti-CRMP2, rabbit anti-ANT 1/2, or rabbit anti-ATP5G1 antibodies, each at a dilution of 1:1000. These blots were generated from a minimum of three separate experiments. The blot membranes were reprobed and Bait Protein Controls were assessed to determine equal immunoprecipitation efficiency. The Input Loading Controls were assessed using rabbit polyclonal anti-VDAC1 antibody (Calbiochem, (San Diego, CA, USA) 1:1000, loading control for CRMP2 input), and mouse monoclonal anti-Complex II 70 kDa subunit antibody (Invitrogen, 1:1000, loading control for ANT1/2 input). The immunoblot images were inverted, and the band densities were measured after subtracting the background using Adobe Photoshop 22.2.0.

### 2.7. Cell Respirometry

A Seahorse XFe24 flux analyzer (Agilent Technologies, Santa Clara, CA, USA) was utilized to measure oxygen consumption rates (OCRs) of cultured cortical neurons (12 DIV) following the manufacturer’s recommendations. Neuronal cultures were seeded into 24-well assay plates at a density of 105 cells per well. Before starting the experiment, the growth medium was replaced with a standard bath solution containing 10 mM glucose and 15 mM pyruvate. This solution consisted of 139 mM NaCl, 3 mM KCl, 0.8 mM MgCl_2_, 1.8 mM CaCl_2_, and 10 mM HEPES, adjusted to pH 7.4. The experiments were conducted at 37 °C.

### 2.8. Mitochondrial Membrane Potential in Cultured Neurons

To assess mitochondrial membrane potential in cultured cortical neurons, we used the fluorescent probe tetramethylrhodamine, methyl ester (TMRM, Thermo Fisher, (Waltham, MA, USA), Cat # T668) [53]. Cells were simultaneously stained with NeuroFluor™ NeuO (Fisher Scientific, (Waltham, MA, USA), Cat # NC1363914) as previously described [54]. After staining, cells were incubated with 20 nM TMRM for 20 min at 37 °C, and TMRM was also included in the bath solution during the experiments. NeuroFluor™ NeuO fluorescence was excited at 480 ± 20 nm and detected through a 505 nm dichroic mirror at 535 ± 25 nm. TMRM fluorescence was excited at 545 ± 15 nm and detected through a 565 nm dichroic mirror at 620 ± 30 nm. In TMRM experiments, excitation of NeuroFluor™ NeuO at 545 ± 15 nm did not produce detectable fluorescence when recorded through a 565 nm dichroic mirror at 620 ± 30 nm. Bright field and fluorescence images were captured using a Nikon Eclipse TE2000-U inverted microscope with a Nikon CFI Plan Apo 100 1.4 NA objective and a Cool SNAPHQ CCD camera (Roper Scientific, Tucson, AZ, USA), controlled by MetaMorph 6.3 software (Molecular Devices, San Jose, CA, USA). TMRM fluorescence analysis was performed as recommended by Connolly et al. [53], with some modifications. Using MetaMorph 6.3 software, uniform regions of interest (ROIs) covering the equal surface area were applied to neuronal cell bodies and intensity of TMRM fluorescence signals was recorded. The background signal was recorded and subtracted from TMRM signal in the cell bodies. Multiple neurons from 5 different platings were analyzed, the processed TMRM signals were pooled together, and the data were presented as arbitrary units (a.u.).

### 2.9. Mitochondrial Superoxide Anion Production in Cultured Neurons

Mitochondrial superoxide anion production was assessed with MitoSOX Red (Molecular Probes) [55]. Simultaneously, cells were co-stained with NeuroFluor™ NeuO as described above. After loading with NeuroFluor™ NeuO, cells were loaded with 2.5 μM MitoSOX Red for 10 min at 37 °C. Fluorescence of NeuroFluor™ NeuO was excited at 480 ± 20 nm and recorded through a 505 nm dichroic mirror at 535 ± 25 nm. Fluorescence of MitoSOX Red was excited at 545 ± 15 nm and recorded through a 565 nm dichroic mirror at 620 ± 30 nm. In experiments with MitoTracker Red, TMRM, and MitoSOX Red, excitation of NeuroFluor™ NeuO at 545 ± 15 nm did not produce measurable fluorescence when we attempted to record through a 565 nm dichroic mirror at 620 ± 30 nm. Bright field and fluorescence images were acquired as described above. MitoSOX Red fluorescence analysis was performed as recommended by Connolly et al. [53], with some modifications, and as described above for TMRM.

### 2.10. Respiration and Membrane Potential in Isolated Mitochondria

Respiration and membrane potential of isolated brain cortical synaptic mitochondria were measured concurrently under uninterrupted stirring in a 0.4 mL chamber with a tightly sealed lid at 37 °C in the incubation medium that included 125 mM KCl, 3 mM KH_2_PO_4_, 0.5 mM MgCl_2_, 10 mM Hepes, pH 7.4, 0.1% BSA free from fatty acids, 10 μM EGTA, 1 mM malate, and 3 mM pyruvate. The incubation chamber was outfitted with a miniature, home-made Clark-type oxygen electrode and a tetraphenylphosphonium (TPP^+^)-sensitive electrode. The slope of the oxygen electrode trace was used to calculate the respiratory rate. Mitochondrial membrane potential was assessed with a TPP^+^-sensitive electrode by following TPP^+^ distribution between the incubation medium and mitochondria [56]. An increase in TPP^+^ concentration outside of mitochondria indicated mitochondrial depolarization, while a decrease in TPP^+^ concentration outside of mitochondria indicated polarization of mitochondria.

### 2.11. Purification and Reconstitution of the ANT

Mitochondria were isolated from brains of ten C57BL/6J mice as we described previously [57,58]. The purification of the ANT from brain mitochondria was performed as described by Gawaz et al. [59] for the ANT from yeast mitochondria and for the ANT from bovine heart mitochondria as described by us [60]. The reconstitution of mouse brain ANT in proteoliposomes was performed as described earlier for the ANT from yeast and bovine heart mitochondria [59,60]. A mixture of 40 mg of phosphatidylcholine (Millipore-Sigma, (Burlington, MA, USA), Cat # 3356) and 1.5 mg of cardiolipin (Millipore-Sigma, Cat # C0563) was dissolved with 0.45 mL of a solution containing 11% detergent C_12_E_8_ (*w*/*v*, Millipore-Sigma, Cat # P8925), 87 mM Na_2_SO_4_, 1 mM EGTA, 175 mM Tricine-OH, pH 8.0, and 87 mM ATP. Then, 0.45 mL of the dissolved lipids were mixed with 1.5 mL of the ANT extract at a final weight ratio of 0.015 protein/phospholipid. This gave a final concentration of 2.5% C_12_E_8_ (*w*/*v*), 20 mM Na_2_SO_4_, 0.23 mM EGTA, 40.4 mM Tricine-OH, pH 8.0, 9.6 mg/mL phospholipid, and 20 mM ATP. Proteoliposomes were formed by slow removal of C_12_E_8_ with ion exchange beads Amberlite XAD-4 (Millipore-Sigma, Cat # XAD4). For the removal of the external solution, the proteoliposomes were passed through Sephadex G-75 (Millipore-Sigma, Cat # G75120) column (30 × 1 cm) pre-equilibrated with 100 mM sucrose, 30 mM Na_2_SO_4_, 1 mM Tricine-OH, pH 7.5, and 1 mM EDTA.

### 2.12. Preparation of Recombinant CRMP2 (rCRMP2)

To produce recombinant CRMP2 protein, we followed established protocols [61]. We transformed BL21 (DE3) Escherichia coli with DNA-encoding sequence-verified pGex-Glu-CaV2.2-type channel constructs for protein expression. Dr. Akihiro Kurimasa from Tottori, Japan, kindly provided the CRMP-2-GST fusion constructs. Protein expression was initiated using 1 mM isopropyl-β-d-thiogalactopyranoside. For purification, the transformed bacteria were grown overnight at 16 °C, then pelleted and lysed in a buffer containing 20 mM Tris (pH 7.5), 200 mM NaCl, 0.1 mM EDTA, 1 mM dithiothreitol, and protease inhibitors. This was carried out using an M-110L microfluidizer from Microfluidics Corp., Newton, MA. The lysate was then treated with Triton X-100 (1% final concentration) and incubated on ice for 30 min. Finally, the mixture was centrifuged at 30,000× *g* for 45 min at 4 °C. The pure CRMP2 protein was concentrated to 30 mg/mL, flash-frozen in liquid nitrogen, and stored at −80 °C in buffer containing 25 mM Tris-HCl, 100 mM glycine, pH 7.3, and 10% glycerol [62].

### 2.13. Evaluation of Adenine Nucleotide Translocase Activity

Brain cortical synaptic mitochondria (30 μg protein), purified using a Percoll gradient, were incubated at 37 °C in 0.4 mL of a medium containing 125 mM KCl, 0.5 mM MgCl2, 3 mM KH_2_PO_4_, 10 mM Hepes (pH 7.4), 0.1% fatty acid-free BSA, 10 µM EGTA, 3 mM pyruvate, and 1 mM malate. The activity of the ANT was evaluated by following ATP efflux from mitochondria in the incubation medium, induced by adding 100 μM ADP as described previously [29,63] with some modifications. ATP in the incubation medium was assessed using the coupled ATP detecting enzymatic system consisting of 2.5 mM glucose, 1.0 U/mL hexokinase (Millipore-Sigma, Cat # H4502), 1.0 U/mL glucose-6-phosphate dehydrogenase (Millipore-Sigma, Cat # G6378), and 0.5 mM NADP^+^ (Millipore-Sigma, Cat # NADP-RO) in the presence of 10 μM P_1_,P_5_-Di(adenosine-5′) pentaphosphate (Ap5A, Millipore-Sigma, Cat # D4022), a specific inhibitor of adenylate kinase [64]. The rate of NADP^+^ reduction and formation of NADPH in the incubation medium is stoichiometrically equivalent to ATP release from mitochondria in the incubation medium mediated by the ANT [29,63]. The formation of NADPH in the incubation medium was followed by measuring NADPH fluorescence (excitation/emission wavelengths 340/460 nm) under gentle stirring with Perkin–Elmer LS 55 luminescence spectrometer equipped with a bio-kinetics accessory. The rate of NADPH formation was determined using the tangent to the initial fragment of the NADPH fluorescence trace and expressed as nmol NADPH × min^−1^ × mg protein^−1^. Control experiments were carried out in the presence of 5 μM carboxyatractyloside (CAT, Millipore-Sigma, Cat # C4992) and 5 μM bongkrekic acid (BKA, Millipore-Sigma, Cat # B6179), the specific ANT inhibitors [24,65,66], to confirm that the ATP release was solely mediated by the ANT.

In experiments with ANT-reconstituted proteoliposomes, 40 μL of proteoliposomes were added to 360 μL of medium containing 100 mM sucrose, 30 mM Na_2_SO_4_, 1 mM Tricine-OH, pH 7.5, and 1 mM EDTA. The ADP/ATP exchange was initiated by adding 100 μM ADP. In some experiments, the ANT proteoliposomes were pretreated with 10 μg/mL of recombinant CRMP2 (rCRMP2) for 5 min under gentle stirring prior to adding ADP. The ATP release from proteoliposomes was measured as described above, but without Ap5A.

### 2.14. Statistics

The experimental data are presented as mean ± SD from the indicated number of experiments. Statistical analyses were performed using unpaired *t*-tests or one-way ANOVA, followed by Bonferroni post hoc tests (GraphPad Prism® version 4.0, GraphPad Software Inc., La Jolla, CA, USA). Each experiment utilized various preparations of isolated mitochondria or cultured neurons.

## 3. Results

### 3.1. CRMP2 Phosphorylation

**Figure 1:**
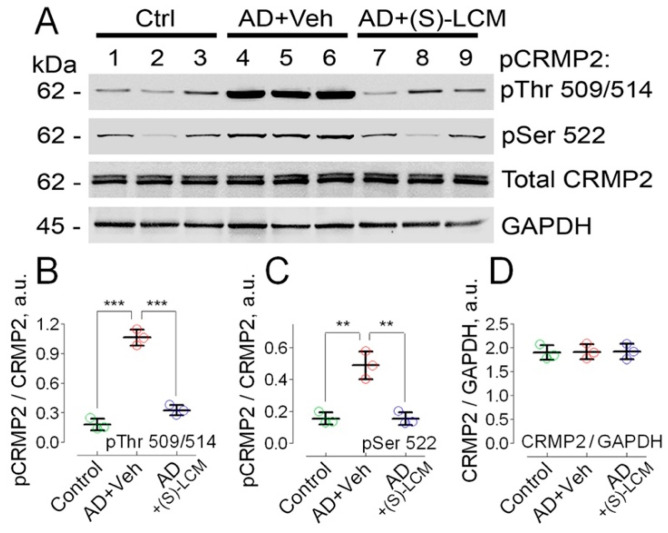
CRMP2 is hyperphosphorylated at Thr 509/514 and Ser 522, but total CRMP2 is unchanged in lysates of brain cortices of 4-month-old APP-SAA KI mice (AD) compared to brain cortices lysates from B6J hAbeta mice (Control, Ctrl). (S)-LCM decreased CRMP2 phosphorylation. In (**A**), lanes 1–3, **Ctrl** mice; lanes 4–6, **AD** mice treated with a vehicle; lanes 7–9, **AD** mice treated with (S)-LCM. (S)-LCM (10 mg/kg body weight) and a vehicle (Veh, 10 μL DMSO in 0.2 mL saline) were delivered by oral gavage for 7 days prior to analysis. In (**B**–**D**), statistical summaries based on densitometry data. Data are mean ± SD. N = 3 biological replicates. ** *p* < 0.01, *** *p* < 0.001.

Previously, it was reported that CRMP2 is hyperphosphorylated at Thr 509/514 and Ser 522 in cultured cortical neurons derived from transgenic APP/PS1 mice [67] and in postmortem brain tissues of AD patients [43,44,45,67]. In the present experiments, we found that CRMP2 was hyperphosphorylated at Thr 509/514 and Ser 522, in lysates of brain cortices of 4-month-old APP-SAA KI mice (Figure 1). The unedited images of immunoblots are shown in Supplemental Figure S1.

**Figure 2:**
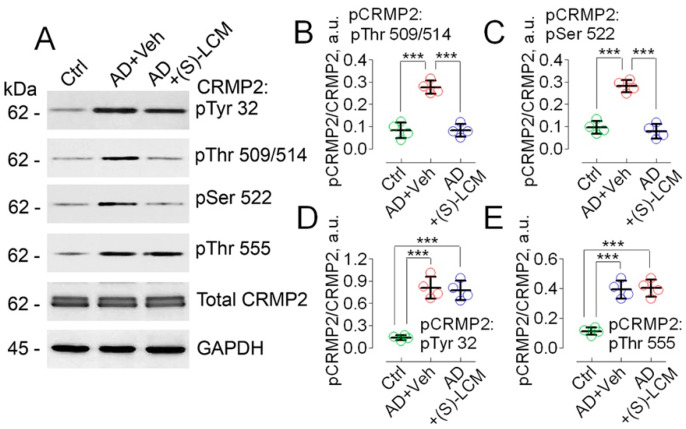
CRMP2 is hyperphosphorylated at Tyr 32, Thr 509/514, Ser 522, and Thr 555 in cultured cortical neurons from APP-SAA KI mice (AD) compared to neurons from B6J hAbeta mice (Control, Ctrl). (S)-LCM prevented CRMP2 hyperphosphorylation at Thr 509/514 and Ser 522, but not at Tyr 32 and Thr 555. Cortical neurons were isolated from P1 AD and Ctrl mice of both sexes and cultured for 12–14 days in vitro (12–14 DIV). In (**A**), representative immunoblots. In (**B**–**E**), statistical summaries based on densitometry data. Where indicated, neurons were treated with either 10 µM (S)-LCM or a vehicle (Veh, 0.01% DMSO) for the last 7 days prior to analysis. GAPDH is a loading control. Data are mean ± SD, N = 4 experiments with cells from different platings. *** *p* < 0.001.

We also found that CRMP2 was hyperphosphorylated at Tyr 32, Thr 509/514, Ser 522, and Thr 555 in cultured cortical neurons from APP-SAA KI mice (Figure 2).

The unedited images of immunoblots are shown in Supplemental Figure S2. To attenuate CRMP2 phosphorylation, we used the small molecule, (S)-LCM. We had previously demonstrated that (S)-LCM binds to CRMP2 and prevents CRMP2 hyperphosphorylation by Cdk5 and GSK-3β kinases, without inhibiting these kinases [68,69]. (S)-LCM delivered to mice by oral gavage (10 mg/kg body weight for 7 days) attenuated CRMP2 phosphorylation in brain cortices of APP-SAA KI mice (Figure 1). In addition, (S)-LCM (10 µM in the growth medium for 7 days prior to experiment) applied to cultured neurons from APP-SAA KI mice reduced CRMP2 phosphorylation at Thr 509/514 and Ser 522, but not at Tyr 32 and Thr 555 (Figure 2).

### 3.2. Cell Respirometry

**Figure 3:**
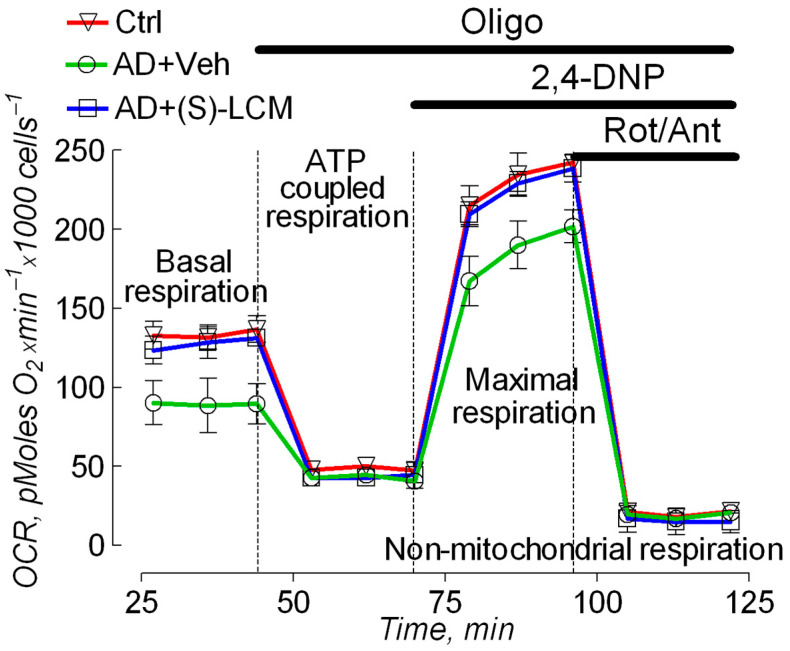
Oxygen Consumption Rate (OCR) of cultured cortical neurons from APP-SAA KI (AD) and B6J hAbeta (Control, Ctrl) mice assessed with Seahorse XFe24 Analyzer, showing (S)-LCM improved respiration in AD neurons. The experiments were performed as we described earlier [54,70,71]. Oligomycin (1 μM, Oligo) inhibits F_1_F_0_-ATP synthase and decreases respiration associated with ATP regeneration; 2,4-dinitorphenol (60 μM, 2,4-DNP) uncouples OXPHOS and induces maximal respiration; rotenone (1 μM, Rot) and antimycin A (1 μM, Ant) are inhibitors of Complexes I and III, respectively, completely inhibit mitochondrial respiration. Where indicated, cells were treated with either a vehicle (Veh, 0.01% DMSO) or 10 µM (S)-LCM for 7 days prior to analysis. Cortical neurons were isolated from P1 AD and Ctrl mice of both sexes and cultured for 14 days in vitro (14 DIV). Data are mean ± SD, N = 5 experiments with neurons from different platings.

To evaluate the functional consequences accompanying alterations in the CRMP2 phosphorylation state, we used cultured cortical neurons from APP-SAA KI and *B6J hAbeta* mice and subjected them to cell respirometry with the Seahorse XFe24 analyzer. In neurons from APP-SAA KI mice, we found a reduction in the rates of basal and maximal respiration, stimulated by the uncoupler 2,4-dinitrophenol (DNP), compared with the respiration of neurons from *B6J hAbeta mice* (Figure 3). Both the basal and the maximal respiration of APP-SAA KI neurons were restored by the pre-treatment of cells with 10 µM (S)-LCM for 7 days prior to the experiment (Figure 3).

### 3.3. Mitochondrial Membrane Potential and Superoxide Production in Cultured Neurons

**Figure 4:**
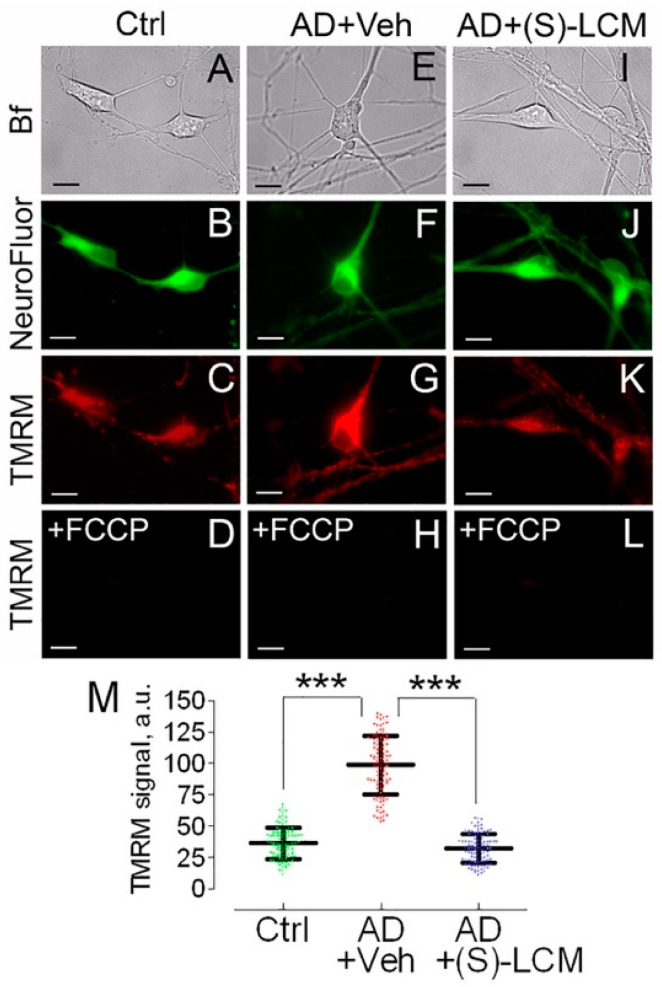
Mitochondrial membrane potential in cultured cortical neurons from APP-SAA KI (AD) and B6J hAbeta (Control, Ctrl) mice, showing (S)-LCM prevented mitochondrial hyperpolarization in AD neurons. Neurons were cultured for 12–14 DIV and then co-stained with TMRM, a mitochondrial membrane potential probe, and NeuroFluor™ NeuO, neuronal marker. The mitochondrial membrane potential was assessed in individual cells by measuring TMRM signal using MetaMorph software [54,78]. The increased TMRM signal indicates higher mitochondrial membrane potential [78]. Bright field (Bf), TMRM, and NeuroFluor images of AD and Ctrl neurons are shown. In (**A**–**D**), cells from Ctrl mice; in (**E**–**H**), cells from AD mice treated with a vehicle (Veh); in (**I**–**L**), cells from AD mice treated with (S)-LCM. Where indicated, cells were treated with either a vehicle (0.01% DMSO) or 10 µM (S)-LCM for 7 days prior to analysis. In (**D**,**H**,**L**), mitochondria were depolarized with 1 μM FCCP as a positive control. In (**M**), statistical summary of TMRM signals. Data are mean ± SD, *** *p* < 0.001, N = 96–102 cells from 5 different platings; a.u., arbitrary units. Scale bars, 10 μm.

The decrease in respiration (Figure 3) could be due to inhibition of the electron transport chain or due to inhibition of the oxidative phosphorylation (OXPHOS) system. If the electron transport chain is inhibited, mitochondria should be depolarized; while, if the OXPHOS system is inhibited, mitochondria should be hyperpolarized. To clarify this, we used TMRM to evaluate mitochondrial membrane potential in cultured cortical neurons from APP-SAA KI and B6J hAbeta mice. Mitochondria in cortical neurons from APP-SAA KI mice were hyperpolarized (Figure 4), consistent with previous findings with mitochondria of *APP^NL-G-F/NL-G-F^* mice, another knock-in AD mouse model [72]. The hyperpolarization of APP-SAA KI mitochondria is consistent with the reduced basal respiration of APP-SAA KI neurons (Figure 3) and is most likely due to suppression of the OXPHOS system as a result of inhibition of the ANT, or F_1_F_0_-ATP synthase, or both.

**Figure 5:**
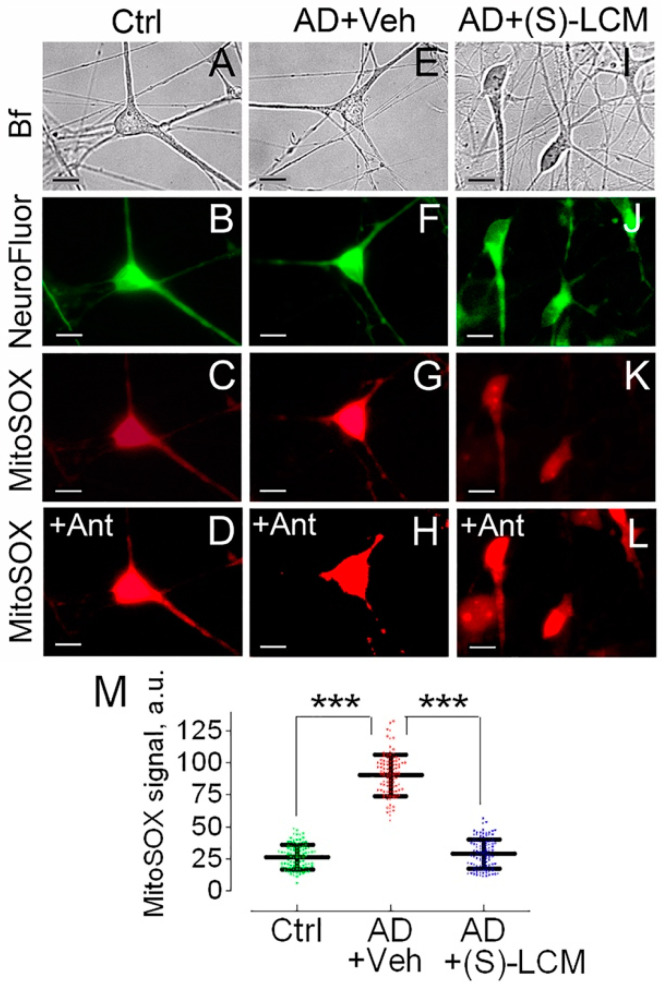
Mitochondrial superoxide anion (O_2_^−^) production in cultured cortical neurons from APP-SAA KI (AD) and B6J hAbeta (Control, Ctrl) mice. (S)-LCM reduced O_2_^−^ production in AD neurons. Neurons were cultured for 12–14 DIV and were detected with NeuroFluor™ NeuO, neuronal marker. O_2_^−^ was evaluated by measuring MitoSOX Red fluorescence using MetaMorph software [54]. The increased MitoSOX signal indicates higher O_2_^−^ level [79]. Bright field (Bf), MitoSOX, and NeuroFluor images of AD and Ctrl neurons are shown. Cells were treated with either a vehicle (Veh, 0.01% DMSO) (**E**–**H**) or 10 µM (S)-LCM (**I**–**L**) for 7 days prior to analysis. In (**A**–**C**,**E**–**G**,**I**–**K**), cells were incubated in the bath solution for 8 min before taking images, and then exposed to 10 μM Antimycin A (Ant) as a positive control for 10 min (**D**,**H**,**L**). In (**M**), statistical summary of MitoSOX signals. Data are mean ± SD, *** *p* < 0.001, N = 95–100 cells from 5 different platings; a.u., arbitrary units. Scale bars, 10 μm.

We also found a higher level of superoxide anion (O_2_^−^) production in mitochondria of cortical neurons from APP-SAA KI mice (Figure 5), in line with the previous reports of elevated ROS production in mitochondria of knock-in *APP^NL-G-F/NL-G-F^* mice [72]. The high O_2_^−^ production in AD mitochondria could be due to elevated mitochondrial membrane potential [73] (Figure 4) and could facilitate an induction of the detrimental permeability transition pore (PTP) in AD mitochondria [74,75,76]. In addition, elevated ROS levels could oxidize CRMP2 and enhance its phosphorylation by GSK-3 [77], creating a self-perpetuating vicious cycle of CRMP2 oxidation and phosphorylation.

### 3.4. Respiration and Membrane Potential in Isolated Mitochondria

**Figure 6:**
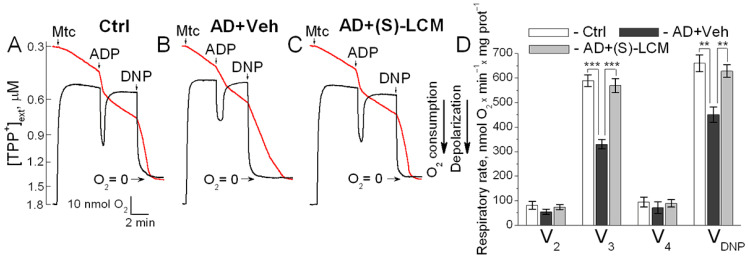
Respiration (red traces) and membrane potential (black traces) of synaptic mitochondria isolated from cortices of 4-month-old APP-SAA KI (AD) and age-matched B6J hAbeta (Control, Ctrl) mice. (S)-LCM improved responses to ADP and 2,4-dinitrophenol (DNP) in AD mitochondria. Mitochondria were isolated from 4-month-old AD and Ctrl mice of both sexes. AD mice were treated with either a vehicle (Veh, 10 µL of DMSO in 0.2 mL saline) or 10 mg (S)-LCM/kg body weight delivered by oral gavage for 7 days before analysis. In (**A**–**C**), representative measurements of mitochondrial respiration and membrane potential. Mitochondria were incubated at 37 °C in KCl-based medium with 1 mM malate plus 3 mM pyruvate [58,81]. Respiration was measured with a Clark-type oxygen electrode, and mitochondrial membrane potential was followed with a tetraphenylphosphonium (TPP^+^)-sensitive electrode [58,81,82]. In (**D**), statistical summary. V_2_, respiration rate before ADP addition; V_3_, respiration stimulated by 300 μM ADP; V_4_, respiration after ADP depletion; V_DNP_, respiration with 60 μM of 2,4-dinitrophenol (DNP). Data are mean ± SD, N = 5 experiments, ** *p* < 0.01, *** *p* < 0.001.

To untangle the mechanisms underlying the decline in the basal and the maximal respiration in APP-SAA KI neurons, we measured oxygen consumption rates and mitochondrial membrane potential in isolated brain cortical synaptic mitochondria (Figure 6). Mitochondria from APP-SAA KI mice accumulated similar amounts of lipophilic cation tetraphenylphosphonium (TPP^+^) compared with *B6J hAbeta* mitochondria, suggesting a similar membrane potential in these mitochondria. The addition of ADP accelerated mitochondrial respiration and transiently depolarized mitochondria. In mitochondria from APP-SAA KI mice, ADP-stimulated respiration was slower, and depolarization was smaller and prolonged (Figure 6). The duration of ADP-induced depolarization, from onset to steady state after repolarization, was 189 ± 6 s in APP-SAA KI mitochondria compared to 124 ± 7 s in B6J hAbeta mitochondria (N = 7, *p* < 0.01). This suggests that either the F_1_F_0_-ATP synthase is inhibited in APP-SAA KI mitochondria as reported earlier [26,27], or ADP enters AD mitochondria slower due to inhibition of the ANT [28,29,30], or both. In addition to ADP-stimulated respiration, the maximal respiration stimulated by DNP was lower with APP-SAA KI mitochondria compared with *B6J hAbeta* mitochondria. Of note, the DNP effect on mitochondrial respiration also depends on ANT activity [80], but not on F_1_F_0_-ATP synthase activity, hence rejecting the hypothesis about F_1_F_0_-ATP synthase involvement in respiratory alterations in AD mitochondria (Figure 6) and AD neurons (Figure 3).

(S)-LCM-attenuated CRMP2 phosphorylation in brain cortices of APP-SAA KI mice (Figure 1) correlated with an improved mitochondrial response to ADP (duration of depolarization: 132.2 ± 8 s, N = 7, *p* < 0.01) and accelerated DNP-stimulated maximal respiration (Figure 6). Thus, the changes in the respiration of isolated mitochondria are consistent with the changes in the respiration of cultured neurons. It is conceivable that CRMP2, in a phosphorylation-dependent manner, modulates ANT-dependent changes in mitochondrial respiration and membrane potential. It is possible that this occurs due to CRMP2 interaction with the ANT and CRMP2-mediated modulation of ANT activity.

### 3.5. CRMP2–ANT Co-Immunoprecipitation

**Figure 7:**
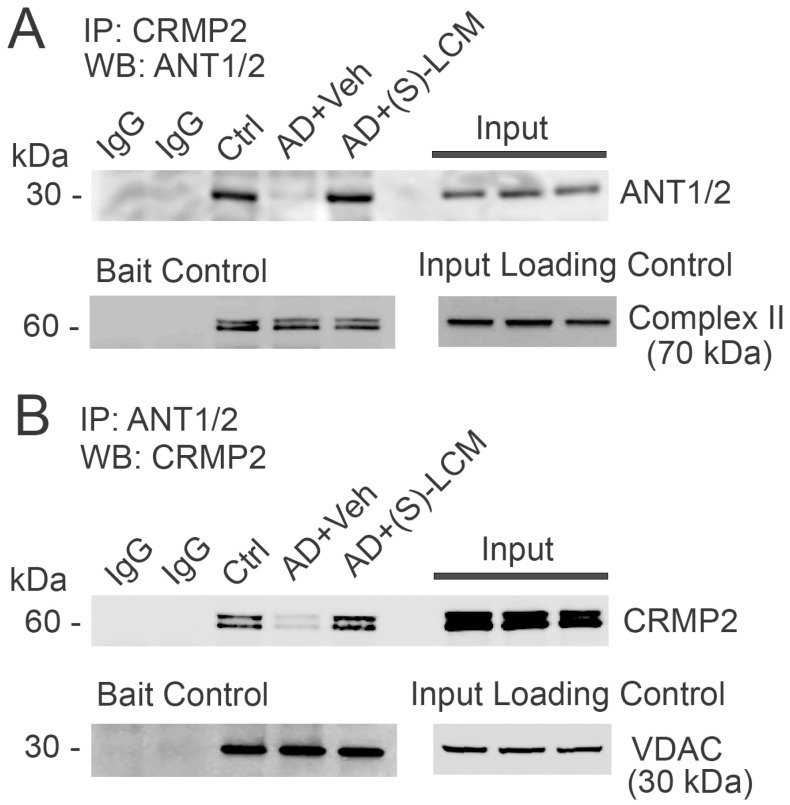
CRMP2 co-immunoprecipitated with the adenine nucleotide translocase (ANT) in brain cortical synaptic mitochondria isolated from 4-month-old B6J hAbeta mice (Control, Ctrl). In mitochondria from 4-month-old APP-SAA KI mice (AD), CRMP2 dissociated from the ANT. (S)-LCM prevented CRMP2 dissociation from the ANT in AD mitochondria. In (**A**,**B**), representative immunoblots with cortical synaptic mitochondria from AD and Ctrl mice. Where indicated, Bait Protein Controls and Input Loading Controls are shown. In (**A**), immunoprecipi-tation was performed with anti-CRMP2 antibody, and the ANT was detected with anti-ANT1/2 antibody. In (**B**), immunoprecipitation was performed with anti-ANT1/2 antibody, and CRMP2 was detected with anti-CRMP2 antibody. Where indicated, AD mice were treated with (S)-LCM (10 mg/kg body weight) or a vehicle (Veh, 10 μL DMSO in 0.2 mL saline), delivered by oral gavage for 7 days prior to analysis. Mitochondria were isolated from mice of both sexes. The Input was 5% of total protein used in pull-down procedure. Representative data from N = 5 biological repeats are shown.

In the following experiments, we investigated the plausible interaction of CRMP2 with the ANT. We detected CRMP2 interaction with the ANT using a co-immunoprecipitation (co-IP) assay applied to cortical synaptic mitochondria isolated from the control, non-AD *B6J hAbeta* mice (Figure 7). This interaction was disrupted in APP-SAA KI mitochondria and rescued by the (S)-LCM pre-treatment of APP-SAA KI mice (Figure 7). The unedited images of immunoblots are shown in Supplemental Figure S3. CRMP2 is hyperphosphorylated in the cortices of APP-SAA KI mice, whereas (S)-LCM prevented CRMP2 hyperphosphorylation (Figure 1), suggesting that CRMP2 interacts with the ANT in a phosphorylation-dependent manner and possibly may modulate ANT activity. We also tested the CRMP2 interaction with *c*-subunits of F_1_F_0_-ATP synthase, exposed to the intermembrane space between the outer and the inner mitochondrial membranes, but did not find evidence for this kind of interaction (Supplemental Figure S4). Therefore, we were focused on the CRMP2–ANT interaction.

### 3.6. ANT Activity in Isolated Mitochondria

**Figure 8:**
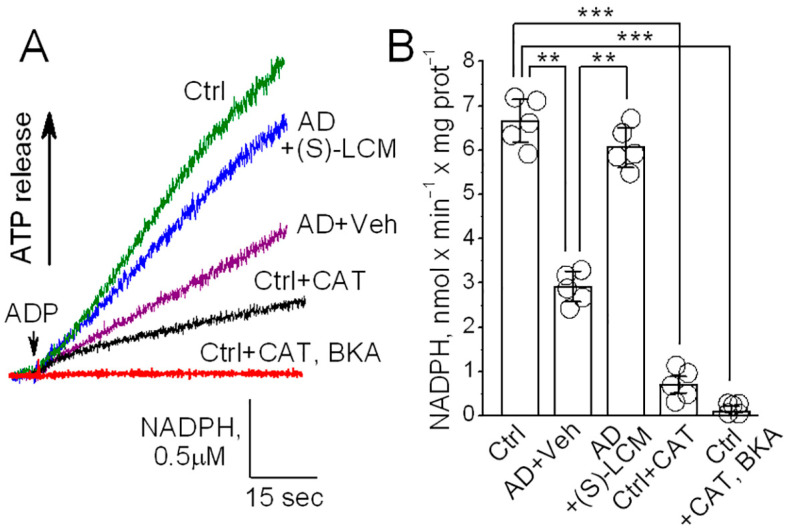
The ANT activity is reduced in synaptic mitochondria isolated from brain cortices of 4-month-old APP-SAA KI (AD) compared with mitochondria from age-matched B6J hAbeta (Control, Ctrl) mice. Pretreatment of AD mice with (S)-LCM improved ANT activity. Where indicated, AD mice were treated with (S)-LCM (10 mg/kg body weight) or a vehicle (Veh, 10 μL DMSO in 0.2 mL saline), delivered by oral gavage for 7 days prior to analysis. As a control, carboxyatractyloside (CAT, 5 μM) and bongkrekic acid (BKA, 5 μM) completely inhibited the ANT. In (**A**), representative fluorescence traces. In (**B**), statistical summary of NADPH measurements. Data are mean ± SD, ** *p* < 0.01, *** *p* < 0.001, N = 5 separate experiments.

To evaluate the functional consequences of CRMP2 interaction with the ANT, we measured ANT transport activity in brain cortical synaptic mitochondria isolated from *B6J hAbeta* and APP-SAA KI mice. We used a previously described method [29,63] with some modifications. This method is based on NADP^+^ reduction and the formation of NADPH in the coupled biochemical reactions catalyzed by hexokinase and glucose-6-phosphate dehydrogenase. In these reactions, formation of NADPH is stoichiometrically equivalent to ATP release from mitochondria, which is then used in these reactions. The ATP release from mitochondria via the ANT-mediated ADP/ATP exchange was initiated by adding 100 μM ADP to mitochondria. The NADPH formation was monitored by following NADPH autofluorescence. The rate of NADPH formation, corresponding to the rate of ATP release from mitochondria, was determined by assessing the tangent to the initial fragment of the NADPH fluorescence signal following ADP addition. Using this method, we found a decrease in the ANT activity in mitochondria from APP-SAA KI mice compared with mitochondria from *B6J hAbeta* mice (Figure 8). The (S)-LCM pre-treatment of APP-SAA KI mice prevented CRMP2 hyperphosphorylation (Figure 1), restored the CRMP2–ANT interaction (Figure 7), and significantly increased the rate of ATP release (Figure 8), suggesting improved ANT activity. Carboxyatractyloside (CAT, 5 μM) and bongkrekic acid (BKA, 5 μM), specific inhibitors of the ANT [24,65,66], completely inhibited NADPH formation, reflecting the suppression of ATP release and indicating that the ANT is solely responsible for the ATP transport. Thus, these results suggest that ANT activity is decreased in APP-SAA KI mitochondria and its activity is correlated with CRMP2 phosphorylation state and CRMP2 binding to the ANT.

### 3.7. ANT Activity in ANT-Reconstituted Proteoliposomes

**Figure 9:**
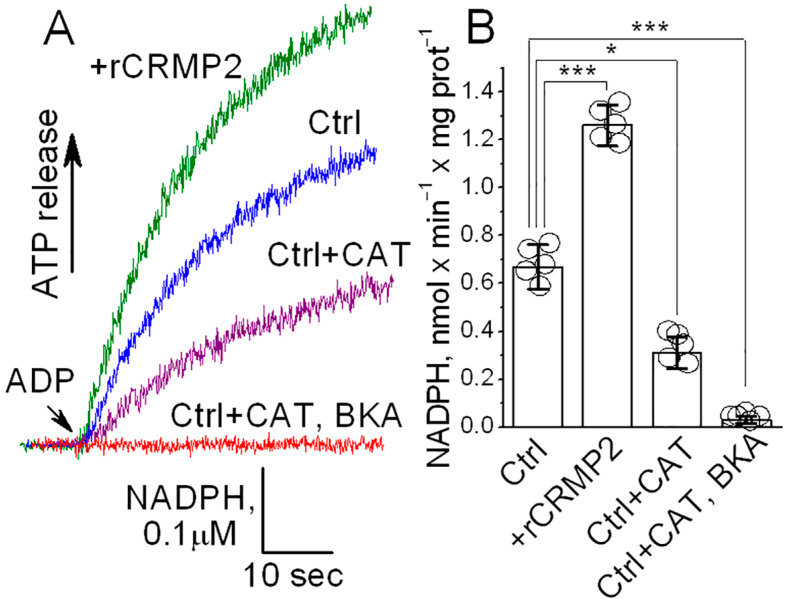
The ANT activity is increased in the ANT proteoliposomes treated with recombinant CRMP2 (rCRMP2). In (**A**), representative fluorescence traces. The ANT proteoliposomes were loaded with 20 mM ATP. Where indicated, recombinant CRMP2 (rCRMP2, 10 μg/mL) was present, and then 100 μM ADP was added. The coupled ATP detecting system consisted of 2.5 mM glucose, 1.0 U/mL hexokinase, 1.0 U/mL glucose-6-phosphate dehydrogenase, 0.5 mM NADP^+^, and 10 μM P_1_,P_5_-Di(adenosine-5′)pentaphosphate, an inhibitor of adenylate kinase. As a control, carboxyatractyloside (CAT, 5 μM) and bongkrekic acid (BKA, 5 μM) completely inhibited the ANT. In (**B**), statistical summary of NADPH measurements. Data are mean ± SD, * *p* < 0.05, *** *p* < 0.001, N = 5 separate experiments.

An inhibition of F_1_F_0_-ATP synthase and reduced ATP production could also contribute to the decreased ATP release from APP-SAA KI mitochondria, measured with the NADP^+^ reduction assay [29,63], which we used in our experiments. Based on experiments with isolated mitochondria (Figure 8), we could not rule out this scenario completely. Thus, whether CRMP2 can directly affect ANT activity remained unclear. Consequently, to clarify this issue, we tested whether CRMP2 selectively modulates ANT activity in experiments with the ANT-reconstituted proteoliposomes. The ANT was purified using hydroxyapatite chromatography and incorporated in the phospholipid liposomes as we described earlier [59,60]. The ANT-proteoliposomes were preloaded with 20 mM ATP. An addition of ADP (100 μM) to ANT-proteoliposomes resulted in ATP release (Figure 9), measured as described above. The pretreatment of the ANT-proteoliposomes with recombinant CRMP2 (rCRMP2, 10 μg/mL for 5 min under gentle stirring prior to adding ADP) accelerated ATP release, whereas CAT (5 μM) and BKA (5 μM) completely inhibited ADP/ATP exchange. These results strongly suggest that CRMP2 stimulates ANT activity, whereas removal of CRMP2 from the ANT is associated with a decrease in ANT transport function.

## 4. Discussion

Alzheimer’s disease (AD) is linked to synaptic dysfunction and neuronal degradation [11,21,22]. Mitochondrial dynamics and bioenergetics defects significantly contribute to these changes [1,2,3,4,5,6,7,8,9,10,11,12,13]. Although the exact mechanisms behind mitochondrial defects in AD are not fully understood, CRMP2 may play a role in modulating mitochondrial functions. Our previous research indicated that CRMP2 regulates mitochondrial dynamics in AD [68]. CRMP2 associates with brain synaptic mitochondria [48,67], with some of it located in the intermembrane space between the outer and inner mitochondrial membranes [48], potentially interacting with various proteins, including ANT [47].

CRMP2 is a cytosolic phosphoprotein that may influence the activity and/or location of different proteins [32]. It is phosphorylated by GSK-3β and Cdk5 kinases [42,83,84,85], which are more active in AD [37,38,39,40]. The hyperphosphorylation of CRMP2 at specific residues is observed in AD brains [41,42,43,44,45] and AD mouse models (APP/PS1 and Tg2576 mice [43,45,46,86]), suggesting its involvement in early AD events [43]. Cortical neurons from wild-type E14 mice, cultured for 3 days and then exposed to the toxic Aβ25–35 peptide fragment for an additional 3 days, show a marked increase in CRMP2 phosphorylation [87]. This suggests that CRMP2 hyperphosphorylation can occur rapidly in cultured cortical neurons following a brief exposure to Aβ25–35. Therefore, our study concentrated on the newly identified role of CRMP2 in mitochondrial function regulation [48,67,88] and its significance in Alzheimer’s disease pathology [67].

In this study, we used brain tissues, cultured cortical neurons, and cortical synaptic mitochondria isolated from APP-SAA KI mice, a knock-in mouse model of AD [49]. The APP-SAA KI mice carry humanized Aβ region R684H, F681Y, and G676R mutations, and the KM670/671NL (Swedish) mutation in exon 16 as well as the E693G (Arctic) and T714I (Austrian) mutations in exon 17 of the mouse *App* gene. At 4 months of age, Aβ deposition and an increased Aβ_42_/Aβ_40_ ratio were detected in APP-SAA KI mice at levels comparable to Tg APP/PS1 mice [49]. The APP-SAA KI mice exhibited increased levels of biomarkers of neurodegeneration, suggesting neuronal degradation [49]. Possible alterations in mitochondrial oxidative metabolism have not been studied with these mice. However, mitochondrial defects were found in *APP^NL-G-F/NL-G-F^* mice, another knock-in APP mouse model [72]. These mice have decreased mitochondrial respiration, reduced ATP production, and increased mitochondrial membrane potential.

The precise mechanisms of CRMP2 involvement in AD pathology have not yet been delineated. In APP/PS1 mice, pioglitazone, a peroxisome proliferator-activated receptor gamma (PPARγ) agonist, decreased CRMP2 phosphorylation, improved energy metabolism, and mitigated motor coordination impairment and long term depression , but it is uncertain whether the effects were due to PPARγ activation or CRMP2 dephosphorylation [86,89]. Conversely, Aβ_25–35_ oligomers induced memory impairment and synaptic plasticity in wild-type mice, but not in phospho-CRMP2-deficient *crmp2^ki/ki^* mice [90], which lacked CRMP2 phosphorylation at Ser 522 and Thr 509/514 due to the Ser 522 replacement with Ala [91]. These results suggest that preventing CRMP2 hyperphosphorylation by Cdk5 and GSK-3β, two kinases activated in AD [37,38,39,40], safeguards against the cognitive decline induced by Aβ_25–35_ oligomers [90]. Thus, CRMP2 hyperphosphorylation may play a crucial role in AD. However, the mechanisms by which CRMP2 contributes to AD pathology have not yet been explored.

In our study, we found that the activity of the ANT is decreased in synaptic mitochondria from APP-SAA KI mice. In experiments with isolated cortical synaptic mitochondria, we found that the decrease in ANT activity correlated with CRMP2 hyperphosphorylation and CRMP2 dissociation from the ANT. (S)-LCM prevented CRMP2 hyperphosphorylation, restored CRMP2 interaction with ANT, and improved ATP transport across the IMM, allowing us to infer the CRMP2-mediated modulation of ANT activity. The stimulating interaction of unphosphorylated CRMP2 with the ANT was further supported by our experiments with rCRMP2 and ANT-proteoliposomes. Importantly, the stimulating interaction of CRMP2 with the ANT might not be unique. Earlier, it was proposed that Bcl-2 interaction with the ANT could stimulate its translocator function, while interaction with the pro-apoptotic protein Bax could diminish the translocator activity [92].

In our experiments, we used the NADP^+^ reduction assay to assess the ANT activity in isolated cortical synaptic mitochondria [29,63]. In contrast to the originally reported method [29], in which the authors measured NADPH spectroscopically by following NADPH absorbance at 340 nm, we measured NADPH autofluorescence to determine the rate of ATP release from mitochondria. This approach appears to be more sensitive than measuring NADPH absorbance [93]. Although NADP^+^ reduction assay is a useful method with which to measure the rate of ATP efflux from mitochondria mediated by the ANT, it cannot completely distinguish between changes in the ANT transport activity and alterations in the F_1_F_0_-ATP synthase enzymatic activity. Indeed, the activities of the F_1_F_0_-ATP synthase and the ANT are decreased in AD [26,27,28,29,30], but the relative significance and mechanisms contributing to these changes are not entirely clear. CRMP2 residing in the intermembrane space could potentially interact not only with the ANT, but also with *c*-subunits of the F_1_F_0_-ATP synthase, facing the intermembrane space. However, in our co-IP experiments, we did not find evidence of CRMP2 interaction with the *c*-subunit of the F_1_F_0_-ATP synthase. Nevertheless, the attenuation of CRMP2 phosphorylation and the enhanced interaction of CRMP2 with the ANT induced by (S)-LCM strongly correlated with the rate of ATP translocation from mitochondria. Thus, it is likely that in our experiments, CRMP2 modulated the ANT activity rather than the activity of the F_1_F_0_-ATP synthase.

This notion is also supported by measurements of neuronal respiration. Whereas the reduction in basal respiration could be due to suppression of ANT transport function or a decrease in F_1_F_0_-ATP synthase activity, the decrease in maximal DNP-stimulated respiration is independent of F_1_F_0_-ATP synthase activity, but depends on activity of the ANT [79]. Preventing CRMP2 hyperphosphorylation with (S)-LCM and restoring the CRMP2–ANT interaction effectively rescued both basal and maximal DNP-stimulated respiration, suggesting that CRMP2 regulates respiration by modulating ANT activity. Interestingly, despite the decreased maximal respiration of APP-SAA KI neurons being significantly higher than the basal respiration rate of the control B6J hAbeta neurons, the sufficient respiratory reserve capacity suggests that this decrease likely does not account for the reduced basal respiration observed in APP-SAA KI mice.

Previous reports indicate that oxidative damage to ANT could reduce its transport activity in AD [29]. Additionally, the inhibition of the mitochondrial voltage-dependent anion channel (VDAC) in AD may weaken the ADP/ATP exchange between the mitochondrial matrix and the cytosol [30]. While these scenarios cannot be ruled out, they may not be the only mechanisms affecting the ADP/ATP exchange in AD. Our data strongly suggest that CRMP2 hyperphosphorylation and its dissociation from ANT may contribute to ANT inactivation in AD. Therefore, our findings highlight the complexity and potential diversity of mechanisms affecting ANT and mitochondrial oxidative metabolism in AD.

There is an apparent discrepancy between mitochondrial hyperpolarization in cultured cortical neurons from APP-SAA KI mice and the lack of such hyperpolarization in mitochondria isolated from these mice. The mitochondrial hyperpolarization, observed with cultured cortical neurons, most likely was due to the partial reduction in ADP/ATP exchange in mitochondria, which continuously occurs in the cell and, most importantly, requires cytosolic ADP. As a result, this led to the partial suppression of oxidative phosphorylation and mitochondrial membrane hyperpolarization. This phenomenon was observed earlier by other investigators [94,95]. In the experiments with isolated organelles, mitochondria were incubated under basal (resting) conditions without ADP that precluded continuous oxidative phosphorylation. Consequently, in these experiments, the partial reduction in ANT activity in mitochondria isolated from APP-SAA KI mice could not affect oxidative phosphorylation and could not influence the mitochondrial membrane potential.

While our study provides strong evidence for the partial reduction in ANT activity in mitochondria of APP-SAA KI mice, we cannot completely rule out the contribution of suppressed F_1_F_o_-ATP synthase activity to the observed mitochondrial phenotype. Indeed, the impaired F_1_F_o_-ATP synthase activity was reported in studies with Aβ peptides and transgenic mouse models of AD and this could contribute to mitochondrial dysfunction in AD [26,27,28,29,30]. However, this issue was not studied with knock-in mouse models of AD. Nevertheless, our new data about the CRMP2-dependent suppression of ANT activity in AD add an important piece of information to our understanding of mitochondrial dysfunction in AD. 

In our previous study, we emphasized CRMP2’s role in regulating mitochondrial dynamics, including changes in morphology and motility, and its potential contribution to neuronal degradation in AD [67]. In this study, we demonstrated that CRMP2 binds to ANT in a phosphorylation-dependent manner and modulates the mitochondrial oxidative metabolism. It is plausible that both alterations in mitochondrial dynamics and changes in oxidative metabolism contribute to mitochondrial defects and subsequent neuronal degeneration in AD. However, the extent to which these mechanisms contribute to neuronal degradation remains uncertain and will be the focus of our future studies.

## Data Availability

The data are contained within the article or Supplementary Materials.

## Supplementary Materials

The following supporting information can be downloaded at: https://www.mdpi.com/article/10.3390/cells14090647/s1, Figure S1. The unedited images of immunoblots for Figure 1A. Figure S2. The unedited images of immunoblots for Figure 2A. Figure S3. The unedited images of immunoblots for Figure 7. Figure S4. The lack of evidence for CRMP2 interaction with *c*-subunits of F_1_F_0_-ATP synthase.
