# Supplementary material for: Collapsin Response Mediator Protein 2 (CRMP2) Modulates Mitochondrial Oxidative Metabolism in Knock-In AD Mouse Model
Source: Cells. 2025 Apr 29;14(9):647. doi: 10.3390/cells14090647 (PMC12071777; doi:10.3390/cells14090647)
Supplement: Supplementary file 1 [file cells-14-00647-s001.zip › cells-3582340-supplementary.pdf]

SUPPLEMENTAL MATERIALS.

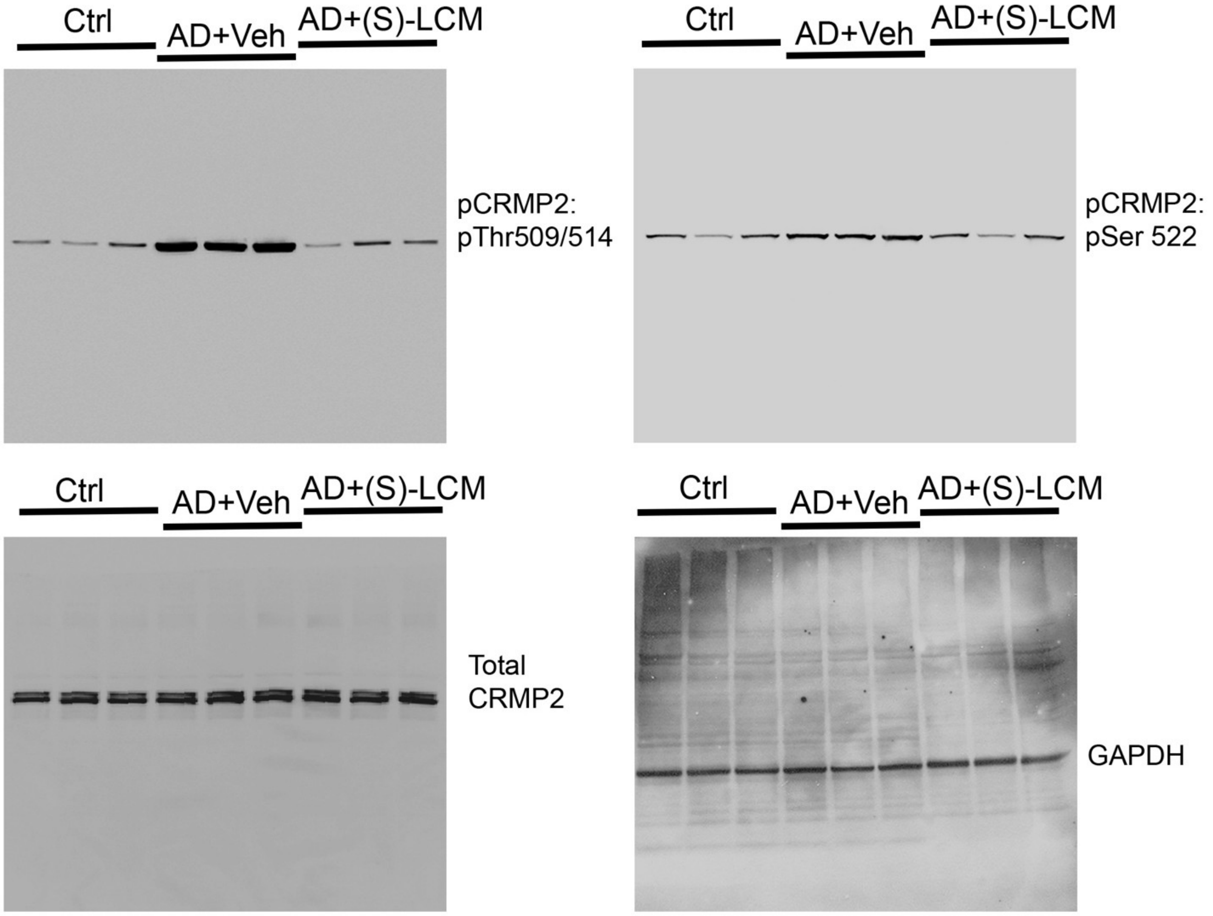

Supplemental Figure S1. The unedited images of immunoblots for Figure 1A.

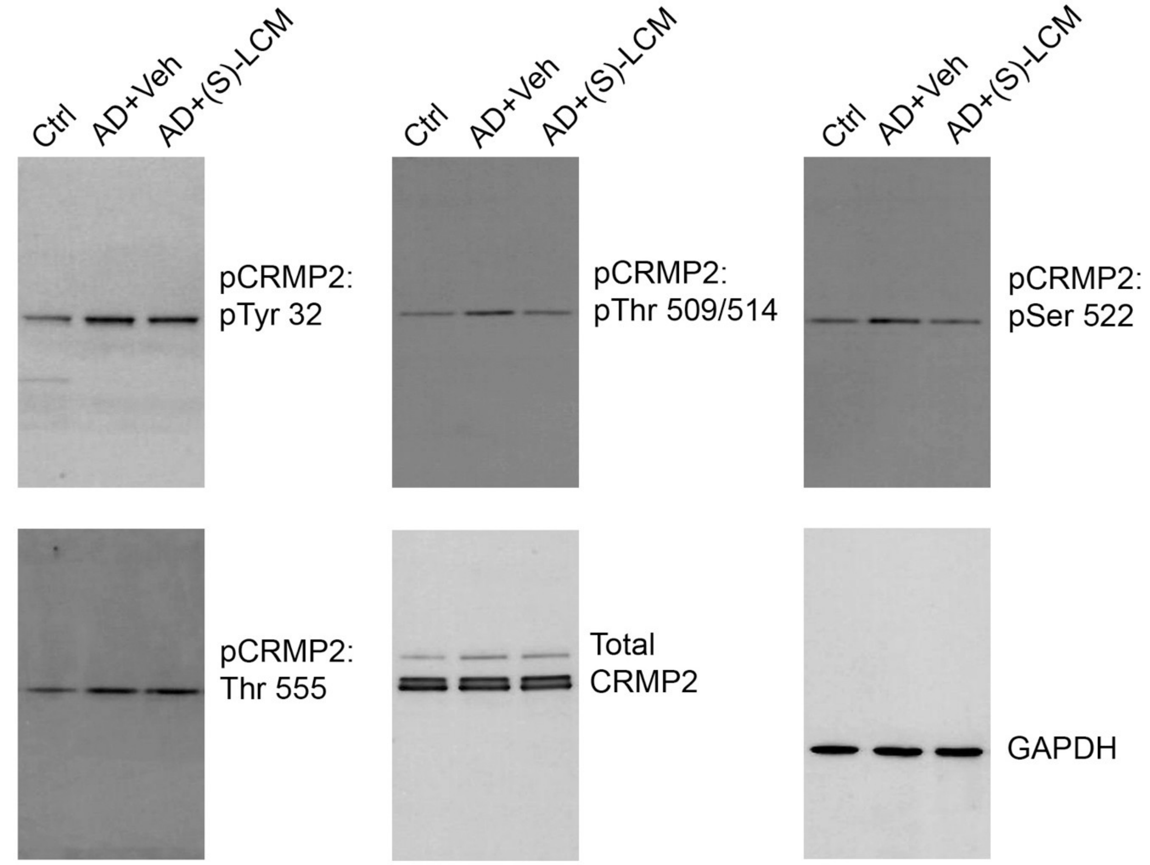

Supplemental Figure S2. The unedited images of immunoblots for Figure 2A.

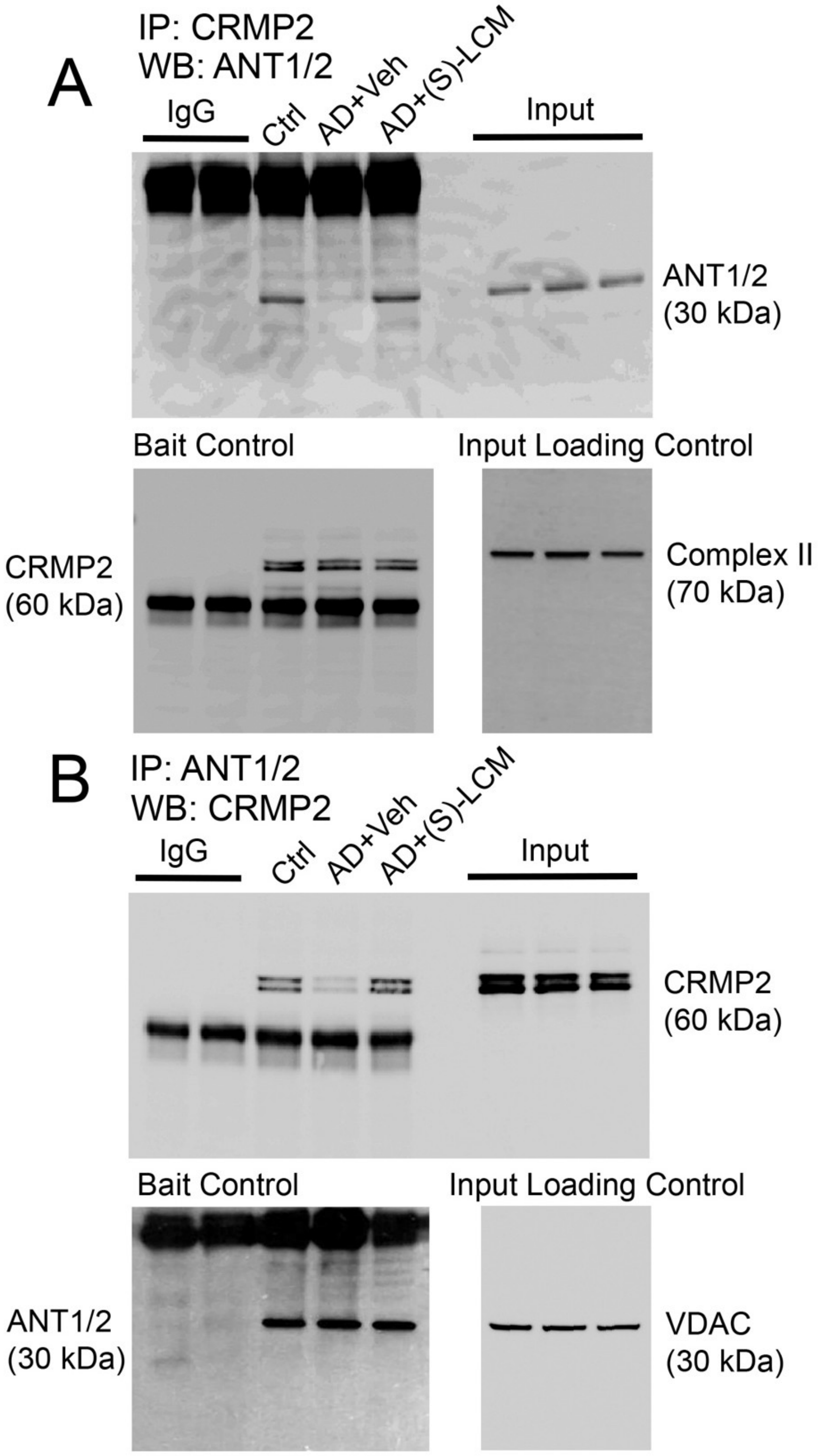

Supplemental Figure S3. The unedited images of immunoblots for Figure 7.

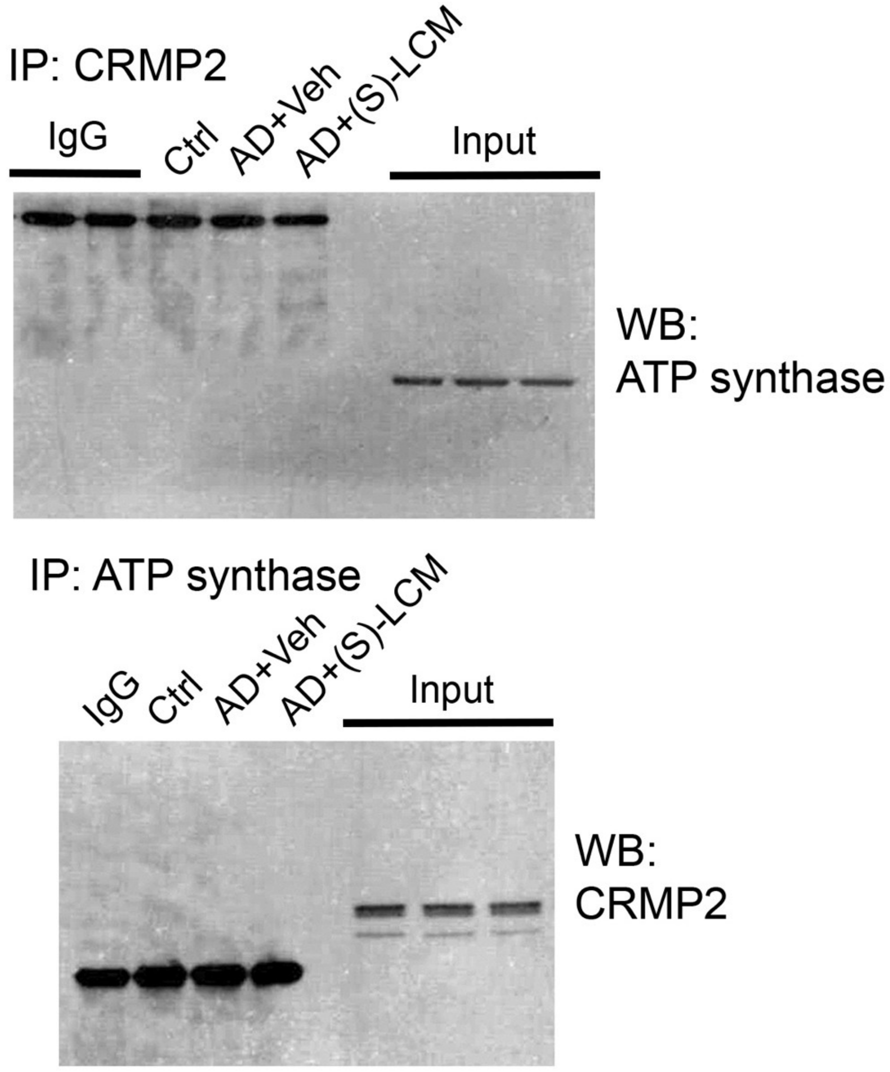

Supplemental Figure S4. The lack of evidence for CRMP2 interaction with *c*-subunits of F_1F_0-ATP synthase.
